# MTHFR C677T genetic polymorphism in combination with serum vitamin B_2_, B_12_ and aberrant DNA methylation of P16 and P53 genes in esophageal squamous cell carcinoma and esophageal precancerous lesions: a case–control study

**DOI:** 10.1186/s12935-019-1012-x

**Published:** 2019-11-12

**Authors:** Da Pan, Ming Su, Guiling Huang, Pengfei Luo, Ting Zhang, Lingmeng Fu, Jie Wei, Shaokang Wang, Guiju Sun

**Affiliations:** 1 Key Laboratory of Environmental Medicine and Engineering of Ministry of Education, and Department of Nutrition and Food Hygiene, School of Public Health, Southeast University, Nanjing, 210009 People’s Republic of China; 2 Huai’an District Center for Disease Control and Prevention, Huai’an, 223200 People’s Republic of China; 3 Jiangsu Vocational College of Medicine, Yancheng, 224005 People’s Republic of China; 4 Jiangsu Provincial Center for Disease Control and Prevention, Nanjing, 210009 People’s Republic of China

**Keywords:** Esophageal squamous cell carcinoma, Esophageal precancerous lesion, Methylenetetrahydrofolate reductase, Vitamin B_2_, Vitamin B_12_, DNA methylation

## Abstract

**Background:**

The study aimed to explore the associations between the interactions of serum vitamin B_2_ or B_12_ levels, aberrant DNA methylation of p16 or p53 and MTHFR C677T polymorphism and the risks of esophageal squamous cell carcinoma (ESCC) and esophageal precancerous lesion (EPL).

**Methods:**

200 ESCC cases, 200 EPL cases and 200 normal controls were matched by age (± 2 years) and gender. Serum vitamin B_2_ and B_12_ levels, MTHFR C677T genetic polymorphisms and the methylation status of genes were assessed. Chi square test, one-way analysis of variance and binary logistic regression were performed.

**Results:**

The lowest quartile of both serum vitamin B_2_ and B_12_ with TT genotype showed significant increased EPL risk (OR = 4.91, 95% CI 1.31–18.35; OR = 6.88, 95% CI 1.10–42.80). The highest quartile of both serum vitamin B_2_ and B_12_ with CC genotype showed significant decreased ESCC risk (OR = 0.16, 95% CI 0.04–0.60; OR = 0.10, 95% CI 0.02–0.46). The ORs of p16 methylation for genotype CT and TT were 1.98 (95% CI 1.01–3.89) and 17.79 (95% CI 2.26–140.22) in EPL, 4.86 (95% CI 2.48–9.50) and 20.40 (95% CI 2.53–164.81) in ESCC, respectively. Similarly, p53 methylation with genotype TT was associated with increased EPL and ESCC risks (OR = 13.28, 95% CI 1.67–105.70; OR = 15.24, 95% CI 1.90–122.62).

**Conclusions:**

The MTHFR C677T genotype and serum vitamin B_2_ or B_12_ levels may interact in ways which associated with the EPL and ESCC risks. The gene–gene interaction suggested that aberrant DNA methyaltion of either p16 or p53 combined with T alleles of MTHFR was associated with increased risks of both EPL and ESCC.

## Background

Esophageal cancer (EC), the eighth commonest cancer and the sixth leading cause of cancer death worldwide, has extremely low 5-year survival rate of approximately 15–25% owing to its aggressive nature and often being diagnosed in late stages [1]. The two histological sub-types of EC, esophageal squamous cell carcinoma (ESCC) and esophageal adenocarcinoma (EAC), have almost completely distinct etiologic and pathologic characteristics, geographic patterns, time trends and major risk factors [2]. ESCC accounts for about 90% cases of EC each year, and it is the predominant type of EC in the developing world, such as in China. It is known that ESCC is associated with a complex combination of genetic, environmental and dietary factors, but many causes of ESCC vary among different regions [2]. Mild, moderate and severe esophageal squamous dysplasia, the recognized esophageal precancerous lesions (EPL) for ESCC, are associated with approximately 3-, 10-, and 30-fold higher risk of ESCC than normal [3–5]. Studies illustrated that some risk or protective factors may be similar for both EPL and ESCC [6].

Deficiencies of some certain micronutrients, such as folate and vitamin B_6_ which are the key methyl donor and cofactor for the enzyme serine hydroxymethyltransferase respectively, have been recognized as the risk factors for cancers including ESCC [7, 8]. Folate and other B vitamins like B_2_, B_6_ and B_12_ play important roles in the one-carbon metabolism pathway, which is associated with DNA methylation, synthesis and impaired DNA repair [9]. 5,10-methylenetetrahydrofolate reductase (MTHFR), the central enzyme in folate metabolism, is involved in the circulation form of folate as it catalyzes the irreversible reduction of 5,10-methylenetetrahydrofolate to 5-methyltetrahydrofolate. Then the catalysis of methionine synthase is responsible for the reaction of 5-methyltetrahydrofolate and homocysteine and the generation of methionine [10]. These cycles require sufficient concentrations of vitamin B_2_ and B_12_ as cofactors for the enzymes MTHFR and methionine synthase, otherwise folate will become trapped as 5,10-methylenetetrahydrofolate or 5-methyltetrahydrofolate and the generation of methionine will be negatively impacted [11]. However, the association between vitamin B_2_ intake and EC risk is not yet entirely understood, and the conclusion has been inconsistent [7]. Several studies illustrated that high intake or plasma concentration of vitamin B_12_ were associated with increased risk of cancers including EC [7, 12–14], but the mechanism remains unclear and epidemiological studies have always showed inconsistent findings [8, 15]. Additionally, nucleotide substitution of C to T at nucleotide 677, the commonest polymorphism for MTHFR gene, results in an alanine to valine replacement so that the activity of MTHFR is reduced [16]. Over the past decade, relations between MTHFR C677T polymorphism and aberrant DNA methylation with the risk of ESCC called for more concern, but the available data remains incomplete and the results are limited [10, 17].

In addition, it is known that carcinogenesis is a multistep process at the genetic level and associated with hundreds of genes thus far. Gene p16 (cyclin dependent kinase inhibitor 2A) and p53 (tumor protein p53) are important tumor suppressor genes located on chromosome 9p21 and 17p13, respectively. They play essential regulatory roles in the G1 cell cycle pathway which is associated with tumorigenesis when becoming dysfunctional [18]. Studies have indicated that p16 methylation and p53 mutations are frequently found in ESCC [19–23], suggesting that genetic and epigenetic alterations in p16 and p53 are involved in the pathogenesis of ESCC [18, 24]. Aberrant DNA promoter hypermethylation in the normal p16 gene was reported to be responsible for the inactivation of p16 gene and the silence of the corresponding gene which is involved in carcinogenesis of esophagus [18]. Meanwhile, it was reported that both p53 mutations and single-nucleotide polymorphism in codon 72 of p53 gene are associated with esophageal carcinogenesis [21–23]. However, the methylation in the p53 promoter in ESCC is rarely studied and gains more concern recently [25].

Our previous study indicated that MTHFR C677T polymorphism may further modify associations between serum concentration of folate and risk of ESCC, but no statistically significant results were found with the risk of EPL [26]. Here, to explore the roles and possible interactions of serum vitamin B_2_ and B_12_ concentrations, MTHFR C677T polymorphism and aberrant DNA methylation of cancer-related genes including p16 and p53 in ESCC and EPL, we carried out a molecular epidemiological study in a Chinese population with extremely high incidence of ESCC.

## Materials and methods

### Study site

Huai’an District, Huai’an City, is an inland rural area in the Northern Jiangsu Province of China with a population with high risk for EC (Additional file 1: Fig. S1). The Center for Disease Control and Prevention (CDC) in Huai’an District established a cancer registry report that the incidence of EC among adults above 40 years old in Huai’an District was 208.09/100,000 from 2008 to 2012, meanwhile, the crude incidence and mortality of EC were 96.15/100,000 and 63.25/100,000, respectively [27]. Our previous studies indicated that environmental exposures, some demographic parameters and genetic polymorphism may play important roles in the esophageal carcinogenesis in this endemic region [27, 28]. Recently, a distinct epidemiological pattern of EPL was observed in Huai’an District. Alcohol drinking plays on a minor role in EPL and excessive smoking shows a significant association. The risk factors which are likely to influence both sexes equally, such as environmental exposures like passive smoking, consuming shallow well water, river water and lake water as the source of drinking water, and dietary factors like frequent intake of pickled food, fried food, hot food, corn and corn flour, and low intake of nuts, bean foods, edible fungi, animal livers, fruits and vegetables, are supposed to take the main responsibility for the development of ESCC [27]. To promote the prevention and definitive treatment of EC and meet the requirements for cost-effectiveness, the government and Cancer Foundation of China have conducted the Early Diagnosis and Early Treatment Project of Esophageal Cancer (EDETPEC) in Huai’an District since 2010. Baseline data and blood samples were collected immediately when participants were enrolled in the project.

### EPL diagnosis

During the EDETPEC, each participant was required to undergo a routine endoscopy examination. 10 ml of 1.2% Lugol’s iodine solution was sprayed on the esophageal mucosa uniformly and then mucosal staining was observed. Normal esophageal mucosa would turn brown (iodine-positive), whereas dysplastic lesions would remain unstained (iodine-negative). Subsequently, unstained tissues were sampled, biopsies were oriented on filter paper, placed in 10% phosphate-buffered formalin and transferred to the pathology laboratory. The biopsies were processed to paraffin blocks, prepared on the slides, then stained with hematoxylin–eosin for histopathological examination. Precancerous lesion for ESCC, which is so called dysplasia, can be classified into mild, moderate and severe dysplasia referring to its histological criteria which were initially illustrated in the 1970s [29, 30] and then modified according to the experience in China [31]. Squamous dysplasia requires the loss of normal cell polarity, the presence of nuclear atypia including nuclear enlargement, pleomorphism and hyperchromatism, and abnormal tissue maturation without invasion of epithelial cells through the basement membrane [6].

### Study population

Between January 2010 and December 2013, 200 diagnosed EPL cases and 200 normal controls aged from 40 to 79 were selected from a total of approximately 5000 residents who had been recruited and undergone a routine endoscopy examination in the EDETPEC. Subsequently, 200 newly diagnosed ESCC cases were selected from Huai’an District cancer registry system during the same period of time. Groups of 200 EPL cases, 200 ESCC cases and 200 normal controls were matched by age (± 2 years) and gender with each other. Individuals who had had a history of cancer, taken vitamin B supplements recently, undergone esophageal cancer surgery, radiotherapy or chemotherapy were not included. The study protocol was approved by the Institutional Review Board of Southeast University Zhongda Hospital with approval number 2012ZDllKY19.0, in accordance with the Declaration of Helsinki. Additionally, all the participants signed informed consent forms.

### Data collection

When obtaining informed consent, trained interviewers collected epidemiological data of socio-demographics, lifestyle, eating habits by face-to-face interviews using a questionnaire. Participants who smoked at least one cigarette or had at least, on average, one alcoholic drink per day continuously for at least 6 months in one’s whole life, were defined as smokers or drinkers. Additionally, 5 ml 12 h fasting blood sample from each participant was collected and centrifuged at 4000 rpm for 5 min to obtain separated serum and leukocyte, then they were stored in the − 80 °C refrigerator immediately.

### Determination of serum vitamin B_2_ and B_12_

Serum vitamin B_2_ and B_12_ levels were determined by double-antibody-sandwich enzyme-linked immunosorbent assay (ELISA). Vitamin B_2_ and B_12_ (human) ELISA kits (Shanghai FanKe industrial Co., Ltd. Shanghai, China) were used in the procedure. All operations were in strict accordance with manufacturer’s instructions. Optical density (OD) values at a wavelength of 450 nm were measured using a multimode microplate reader (Mithras LB 940, Berthold Technologies, Bad Wildbad, Germany), and then concentrations of serum vitamin B_2_ and B_12_ were calculated accordingly.

### Detection of MTHFR polymorphism and DNA methylation

Genomic DNA was extracted from leukocyte samples by using Wizard^®^ Genomic DNA Purification kit (A1120, Promega, WI, USA). MTHFR C677T genetic polymorphisms were assessd by polymerase chain reaction-restriction fragment length polymorphism (PCR-RFLP) with the sense primer 5′-CGA AGC AGG GAG CTT TGA GGC TG-3′ and the antisense primer 5′-AGG ACG GTG CGG TGA GAG TG-3′. A PCR premix kit (SK2072, Sangon, Shanghai, China) was used to conduct PCR amplification, and the process was carried out in an automated themocycler (AG6321, Eppendorf, Hamburg, Germany). The PCR condition was initial denaturation at 94 °C for 5 min, followed by 35 cycles of denaturation at 94 °C for 30 s, annealing at 67 °C for 30 s, extension at 72 °C for 60 s, and final extension at 72 °C for 10 min. PCR products were digested overnight at 37 °C with restriction endonuclease *Hin*f I (R0155 V, New England Biolabs, UK) for recognizing and cutting the variant type sequence. The digestive PCR products were resolved on 3% agarose gel and visualized under UV illumination after being stained with ethidium bromide (Additional file 2: Fig. S2(a)). The wild genotype CC has one band of 233 bp. The heterozygote CT has three bands of 233, 176 and 57 bp, and the variant genotype TT has two bands of 176 and 57 bp.

The methylation status at the promoter region of gene p16 and p53 was assessd by methylation-specific PCR (MSP) after sodium bisulfate modification of DNA [32]. The primers used for p16 were methylated sense primer 5′-TTA TTA GAG GGT GGG GCG GAT CGC-3′ and antisense primer 5′-GAC CCC GAA CCG CGA CCG TAA-3′, and unmethylated sense primer 5′-TTA TTA GAG GGT GGG GTG GAT TGT-3′ and antisense primer 5′-CAA CCC CAA ACC ACA ACC ATA A-3′. The primers used for p53 were methylated sense primer 5′-GTA GTT TGA ACG TTT TTA TTT TGG C-3′ and antisense primer 5′-CCT ACT ACG CCC TCT ACA AAC G-3′, and unmethylated sense primer 5′-GTA GTT TGA ATG TTT TTA TTT TGG T-3′ and antisense primer 5′-CCT ACT ACA CCC TCT ACA AAC A-3′. PCR products were loaded onto 2% agarose gel and visualized after being stained with ethidium bromide (Additional file 2: Fig. S2(b)).

### Statistical analysis

Baseline data were double-entered and validated in an established database with Epidata version 3.1 and then processed in Microsoft Excel. Statistical analyses were performed using the statistical package SPSS version 17 (SPSS, Chicago, IL, USA). Differences in the serum vitamin B_2_ and B_12_ levels, distribution of socio-demographic characteristics, lifestyle, methylation and genotype frequencies among controls, EPL and ESCC cases were evaluated using Kruskal–Wallis test, Mann–Whitney U test, Chi square (χ^2^) test and one-way analysis of variance (ANOVA), wherever appropriate. The continuous variables of serum vitamin B_2_ and B_12_ levels were categorized into quartiles (Q1, Q2, Q3 and Q4) based on the levels of normal controls. Binary logistic regression was performed to assess the association between serum vitamin B_2_ or B_12_ level and risk of EPL or ESCC. The effects of interaction of serum vitamin B_2_ or B_12_ level and MTHFR C667T genotype on the risk of EPL and ESCC, and the joint effects of MTHFR C667T genotype and DNA methylation on the risks of EPL and ESCC were evaluated using a multiplicative interaction model based on logistic regression. Analyses were adjusted for confounding variables including gender, age, tobacco use and alcohol use. Results were expressed by calculated odds ratio (OR) and its corresponding 95% confidence interval (CI), and statistical significance was considered as p < 0.05 (two-tailed).

## Results

### Socio-demographic characteristics of the subjects

In this study, 200 normal controls, 200 diagnosed EPL cases and 200 newly diagnosed ESCC cases with mean age of 62.69 ± 5.37, 61.29 ± 6.60 and 62.28 ± 6.49 years were enrolled and required to be age- (± 2 years) and gender-matched. In all, 106 pairs were males and 94 pairs were females. As shown in Table 1, ANOVA indicated that there was no statistical significance in age among three groups (*p *> 0.05).

**Table 1:** Age characteristics of participants (years, mean ± SD)

Category	Control (n = 200)	EPL (n = 200)	ESCC (n = 200)	*p* value
Age range	43–76	45–77	45–77	
Male	62.89 ± 5.10	61.26 ± 6.82	62.57 ± 6.65	0.133
Female	62.46 ± 5.68	61.31 ± 6.38	61.94 ± 6.34	0.439
Total	62.69 ± 5.37	61.29 ± 6.60	62.28 ± 6.49	0.066

*SD* Standard deviation

### Serum vitamin B_2_ and B_12_ levels of the subjects

**Fig. 1:**
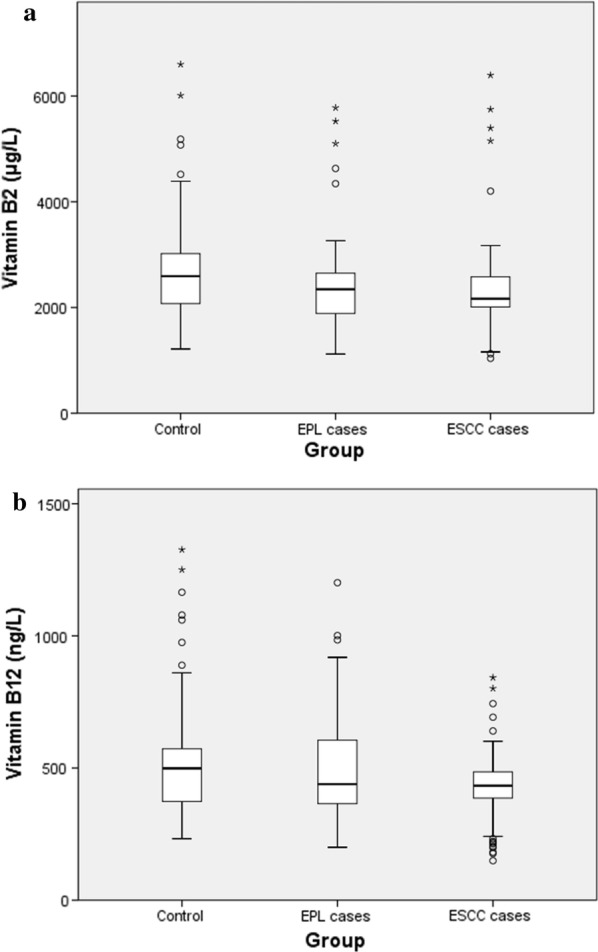
**a** Box and whiskers plot of serum vitamin B_2_ level in three groups. **b** Box and whiskers plot of serum vitamin B_12_ level in three groups. The box values range from 25 to 75 percentiles of 200 subjects; the line within the box represents the median. The T-shaped bars at both sides of the box represent values ranging from 5 to 25 and 75 to 95 percentiles; the dots represent outliers

The distributions of serum vitamin B_2_ and B_12_ levels in normal controls, EPL cases and ESCC cases are shown in Table 2 and Fig. 1. Kruskal–Wallis test indicated that there were statistical significances in both vitamin B_2_ and B_12_ among three groups (*p *< 0.01). In addition, compared with control group, both EPL and ESCC groups showed statistical significances in vitamin B_2_ level, and only ESCC group showed statistical significance in vitamin B_12_ level (*p *< 0.001, Mann–Whitney U test). However, no statistical significance was found in both vitamin B_2_ and B_12_ between EPL group and ESCC group (*p *> 0.05, Mann–Whitney U test).

**Table 2:** Serum vitamin B_2_ and B_12_ levels in three groups ((Median (25th–75th))

Category	Control (n = 200)	EPL (n = 200)	ESCC (n = 200)	*p* value^a^
Vitamin B_2_ (μg/l)	2592.69 (2074.39–3019.57)	2344.65 (1887.37–2648.32)^b^	2165.86 (2010.11–2577.37)^b^	< 0.001
Vitamin B_12_ (ng/l)	499.07 (375.09–570.88)	438.91 (365.82–605.93)	433.07 (386.83–485.20)^b^	0.002

^a^*p* value of Kruskal–Wallis test among the three groups

^b^Compared with control group, *p *< 0.001 (Mann–Whitney U test)

### The association between serum vitamin B_2_ and B_12_ levels and risk of EPL and ESCC

Serum vitamin B_2_ and B_12_ concentrations were classified into quartiles. As shown in Table 3, results indicated that the highest quartile of serum vitamin B_2_ was inversely associated with the risk of both EPL (OR = 0.24, 95% CI 0.11–0.51) and ESCC (OR = 0.19, 95% CI 0.08–0.44) when compared with the lowest quartile. The highest quartile of serum vitamin B_12_ was inversely associated with the risk of ESCC (OR = 0.18, 95% CI 0.07–0.42) when compared with the lowest quartile. However, the second lowest quartiles of both vitamin B_2_ and B_12_ were associated with the increased risk of ESCC when compared with the lowest quartiles (OR = 1.90, 95% CI 1.10–3.26; OR = 1.87, 95% CI 1.08–3.24).

**Table 3:** Associations between serum vitamin B_2_ and B_12_ levels and risk of EPL and ESCC

	Quartile				
	Q1	Q2	Q3	Q4	
Esophageal precancerous lesions					
Vitamin B_2_					
Concentration range, μg/l	< 2074.39	2074.39–2592.69	2592.69–3019.57	> 3019.57	
No. of cases/controls	67/50	68/50	43/50	22/50	
Adjusted OR (95% CI)^a^	1.00 (reference)	0.62 (0.34–1.13)	0.64 (0.35–1.16)	0.24 (0.11–0.51)	
*p* value		0.121	0.140	< 0.001	
Vitamin B_12_					
Concentration range, ng/l	< 375.09	375.09–499.07	499.07–570.88	> 570.88	
No. of cases/controls	53/50	68/50	21/50	58/50	
Adjusted OR (95% CI)^a^	1.00 (reference)	1.01 (0.53–1.91)	0.54 (0.26–1.13)	1.32 (0.70–2.49)	
*p* value		0.980	0.104	0.396	
Esophageal squamous cell carcinoma					
Vitamin B_2_					
Concentration range, μg/l	< 2074.39	2074.39–2592.69	2592.69–3019.57	> 3019.57	
No. of cases/controls	51/50	104/50	37/50	8/50	
Adjusted OR (95% CI)^a^	1.00 (reference)	1.90 (1.10–3.26)	0.75 (0.41–1.37)	0.19 (0.08–0.44)	
*p* value		0.021	0.345	< 0.001	
Vitamin B_12_					
Concentration range, ng/l	< 375.09	375.09–499.07	499.07–570.88	> 570.88	
No. of cases/controls	49/50	115/50	27/50	9/50	
Adjusted OR (95% CI)^a^	1.00 (reference)	1.87 (1.08–3.24)	0.55 (0.28–1.05)	0.18 (0.07–0.42)	
*p* value		0.025	0.071	< 0.001	

^a^Adjusted for gender, age, tobacco smoking and alcoholic drinking

### MTHFR C677T polymorphism and DNA methylation in the study subjects

The proportions of CC, CT and TT genotypes were 49.5%, 41.0% and 9.5% in control group, 42.5%, 40.0% and 17.5% in EPL group, and 42.0%, 48.5% and 9.5% in ESCC group, respectively (Table 4). Hardy–Weinberg calculation was performed for control group, and no significant deviation from the Hardy–Weinberg expectation was observed (χ^2^ = 0.11, *p *= 0.74). Compared with control group, EPL group had a higher proportion of variant genotype TT and the difference was statistically significant (χ^2^ = 5.79, *p *< 0.05). The frequency of T allele in EPL group was higher than that in control group as well (χ^2^ = 5.03, *p *< 0.05). However, no statistically significant difference was found in the distribution of CT, TT genotypes and T allele between ESCC and control groups (*p *> 0.05). Additionally, our finding of Chi square test showed that p16 methylation was associated with 58% and 216% increases in the risk of EPL and ESCC, respectively (*p *< 0.05). Meanwhile, p53 methylation was associated with 121% increase in the risk of ESCC (*p *< 0.05).

**Table 4:** Genotype and allele frequencies for the MTHFR C677T polymorphism and DNA methylation in EPL cases, ESCC cases and healthy controls

Variables	Control n (%)	EPL			ESCC		
		n (%)	OR (95% CI)	*p* value	n (%)	OR (95% CI)	*p* value
MTHFR C677T							
CC	99 (49.5)	85 (42.5)	1.00		84 (42.0)	1.00	
CT	82 (41.0)	80 (40.0)	1.14 (0.74–1.73)	0.55	97 (48.5)	1.39 (0.92–2.11)	0.12
TT	19 (9.5)	35 (17.5)	2.15 (1.14–4.03)	0.02	19 (9.5)	1.18 (0.59–2.37)	0.65
C	280 (70.0)	250 (62.5)	1.00		265 (66.3)	1.00	
T	120 (30.0)	150 (37.5)	1.40 (1.04–1.89)	0.03	135 (33.8)	1.19 (0.88–1.60)	0.26
DNA methylation							
p16 methylation	70 (35.0)	92 (46.0)	1.58 (1.06–2.37)	0.03	126 (63.0)	3.16 (2.10–4.76)	< 0.01
p53 methylation	74 (36.5)	84 (42.0)	1.23 (0.83–1.84)	0.36	113 (56.5)	2.21 (1.48–3.30)	< 0.01

Based on the significant results of the comparison of MTHFR C677T genotype and allele frequencies between EPL and controls provided by Tables 4 and 5 further illustrated the internal comparison of genotype and allele frequencies for the MTHFR C677T polymorphism among mild, moderate and severe EPL cases by Chi square test. There was no statistically significant difference between mild and moderate EPL cases, whereas TT genotype and T allele were more frequent in severe EPL cases than in mild EPL cases (OR = 4.19, 95% CI 1.41–12.44; OR = 2.33, 95% CI 1.29–4.21).

**Table 5:** Genotype and allele frequencies for the MTHFR C677T polymorphism in mild, moderate and severe EPL cases

MTHFR C677T genotype frequencies	Mild EPL n (%)	Moderate EPL			Severe EPL		
		*n* (%)	OR (95% CI)	*p* value	*n* (%)	OR (95% CI)	*p* value
CC	49 (50.5)	27 (37.0)	1.00		9 (30.0)	1.00	
CT	35 (36.1)	34 (46.6)	1.76 (0.91–3.43)	0.09	11 (36.7)	1.71 (0.64–4.57)	0.28
TT	13 (13.4)	12 (16.4)	1.68 (0.67–4.18)	0.27	10 (33.3)	4.19 (1.41–12.44)	0.01
C	133 (68.6)	88 (60.3)	1.00		29 (48.4)	1.00	
T	61 (31.5)	58 (39.7)	1.44 (0.92–2.25)	0.11	31 (51.7)	2.33 (1.29–4.21)	0.01

### Interaction between serum vitamin B_2_, B_12_ and MTHFR C677T polymorphism

The interaction between serum vitamin B_2_, B_12_ and MTHFR C677T genotype was determined by logistic regression analysis using controls, EPL and ESCC cases. As shown in Table 6, after adjustment for gender, age, tobacco smoking and alcoholic drinking, the interaction of the lowest quartile of both serum vitamin B_2_ and B_12_ with MTHFR C677T genotypes in EPL showed significant increased risk for variant genotype TT (OR = 4.91, 95% CI 1.31–18.35; OR = 6.88, 95% CI 1.10–42.80). The interaction of the highest quartile of both serum vitamin B_2_ and B_12_ with MTHFR C677T genotypes in ESCC showed significant decreased risk for wild genotype CC (OR = 0.16, 95% CI 0.04–0.60; OR = 0.10, 95% CI 0.02–0.46), as well as for heterozygote CT (OR = 0.23, 95% CI 0.07–0.77; OR = 0.18, 95% CI 0.05–0.62). Interestingly, heterozygote CT along with the second lowest quartile of both serum vitamin B_2_ and B_12_ conferred an approximately twofold increased ESCC risk (OR = 2.45, 95% CI 1.13–5.30; OR = 2.38, 95% CI 1.10–5.17), and the results of vitamin B_2_ and B_12_ showed similarity to some degree. However, the variant genotype TT along with the highest quartile of serum vitamin B_12_ conferred a tenfold increased EPL risk (OR = 10.08, 95% CI 2.22–45.71).

**Table 6:** Association between vitamin B_2_, B_12_ with the MTHFR C677T genotype and the risk of EPL and ESCC

Variables	EPL [OR (95% CI)^a^, *p* for interaction]			ESCC [OR (95% CI)^a^, *p* for interaction]		
	CC	CT	TT	CC	CT	TT
Vitamin B_2_ (μg/l)						
Q1 (< 2074.39)	1.00 (reference)	2.05 (0.85–4.99) 0.112	4.91 (1.31–18.35) 0.018	1.00 (reference)	1.13 (0.47–2.68) 0.790	1.25 (0.28–5.63) 0.771
Q2 (2074.39–2592.69)	1.16 (0.45–3.02) 0.760	0.85 (0.32–2.26) 0.750	1.56 (0.47–5.22) 0.469	2.13 (0.95–4.76) 0.067	2.45 (1.13–5.30) 0.023	0.78 (0.22–2.76) 0.702
Q3 (2592.69–3019.57)	0.60 (0.23–1.57) 0.294	1.79 (0.70–4.56) 0.226	2.98 (0.62–14.36) 0.174	0.54 (0.23–1.30) 0.171	1.10 (0.44–2.74) 0.833	1.67 (0.33–8.32) 0.534
Q4 (> 3019.57)	0.07 (0.01–0.61) 0.016	0.49 (0.16–1.49) 0.206	2.23 (0.51–9.69) 0.284	0.16 (0.04–0.60) 0.006	0.23 (0.07–0.77) 0.017	0.31 (0.03–3.02) 0.315
Vitamin B_12_ (ng/l)						
Q1 (< 375.09)	1.00 (reference)	2.03 (0.70–5.87) 0.193	6.88 (1.10–42.80) 0.039	1.00 (reference)	0.93 (0.38–2.28) 0.868	1.65 (0.25–10.91) 0.603
Q2 (375.09–499.07)	1.18 (0.41–3.38) 0.760	2.06 (0.75–5.69) 0.162	3.30 (0.79–13.88) 0.103	1.49 (0.69–3.25) 0.313	2.38 (1.10–5.17) 0.028	1.10 (0.28–4.37) 0.892
Q3 (499.07–570.88)	0.74 (0.23–2.37) 0.612	0.73 (0.19–2.88) 0.657	1.83 (0.49–6.81) 0.365	0.42 (0.16–1.09) 0.075	0.73 (0.27–2.00) 0.545	0.49 (0.13–1.84) 0.290
Q4 (> 570.88)	1.32 (0.45–3.88) 0.620	2.41 (0.89–6.53) 0.085	10.08 (2.22–45.71) 0.003	0.10 (0.02–0.46) 0.003	0.18 (0.05–0.62) 0.006	0.73 (0.11–4.85) 0.748

^a^Adjusted for gender, age, tobacco smoking and alcoholic drinking

### Interaction between DNA methylation and MTHFR C677T polymorphism

Results were adjusted for potential confounders including gender, age, tobacco smoking and alcoholic drinking. The methylation statuses of p16 and p53 in EPL and ESCC cases were significantly associated with MTHFR C677T genotypes. Compared with the wild genotype CC, no statistically significant difference was found in genotype CT or TT in both EPL and ESCC when p16 or p53 was unmethylated (Table 7). However, the ORs of p16 methylation for genotype CT and TT were 1.98 (95% CI 1.01–3.89) and 17.79 (95% CI 2.26–140.22) in EPL, 4.86 (95% CI 2.48–9.50) and 20.40 (95% CI 2.53–164.81) in ESCC, respectively. Similarly, p53 methylation with variant genotype TT was associated with increased risks of EPL and ESCC (OR = 13.28, 95% CI 1.67–105.70; OR = 15.24, 95% CI 1.90–122.62).

**Table 7:** Association between DNA methylation with the MTHFR C677T genotype and the risk of EPL and ESCC

DNA methylation	EPL [OR (95% CI)^a^, *p* for interaction]			ESCC [OR (95% CI)^a^, *p* for interaction]		
	CC	CT	TT	CC	CT	TT
p16U	1.00 (reference)	0.88 (0.50–1.53) 0.640	1.32 (0.62–2.80) 0.474	1.00 (reference)	1.03 (0.56–1.90) 0.921	0.66 (0.25–1.77) 0.409
p16 M	1.04 (0.58–1.85) 0.900	1.98 (1.01–3.89) 0.048	17.79 (2.26–140.22) 0.006	1.91 (1.95–3.47) 0.033	4.86 (2.48–9.50) < 0.001	20.40 (2.53–164.81) 0.005
p53U	1.00 (reference)	0.90 (0.46–1.40) 0.441	1.25 (0.59–2.63) 0.558	1.00 (reference)	0.90 (0.50–1.60) 0.710	0.49 (0.19–1.31) 0.156
p53 M	0.77(0.43–1.37) 0.369	1.56(0.79–3.09) 0.205	13.28(1.67–105.70) 0.015	1.15(0.64–2.06) 0.643	3.27(1.68–6.34) < 0.001	15.24(1.90–122.62) 0.010

*U* unmethylated, *M* methylated

^a^Adjusted for gender, age, tobacco smoking and alcoholic drinking

## Discussion

In this study, we tried to evaluate the possible association and interaction of epigenetics and genetics, serum vitamin B_2_ and B_12_ levels and the risks of EPL and ESCC in the high-incidence area of Northern Jiangsu Province of China. Our study suggested that: firstly, healthy controls were more likely to have higher levels of vitamin B_2_ and B_12_ than ESCC cases; secondly, variant genotype TT and the T allele were associated with significantly increased risk of EPL; thirdly, the MTHFR C677T genotype may modify association between serum vitamin B_2_ or B_12_ levels and the risks of EPL and ESCC; fourthly, gene–gene interaction was observed as there were strong association between the interaction of p16 and p53 methylations and MTHFR C677T polymorphism and the risks of EPL and ESCC.

To the best of our knowledge, the MTHFR C677T variant is a C to T transition in exon 4 at nucleotide 677 and results in an alanine to valine replacement at position 222 of the MTHFR amino acid sequence [33]. Compared with homozygous wild genotype CC, the activity of MTHFR was observed to reduce by 35% and 70% for heterozygote CT and variant genotype TT, respectively [34]. Previous study also indicated that genetic polymorphisms of enzymes involved in folate metabolism might have an influence on neoplasia in the esophagus, and TT genotype is usually along with lower serum folate levels and higher homocysteine levels than homozygous wild genotype [35, 36].

Individually, both the serum levels of vitamin B_2_ and B_12_ and the MTHFR C677T genotypes may be associated with the risks of EPL and ESCC. However, the serum levels of vitamin B_2_ and B_12_ and the MTHFR C677T genotypes interact in ways which has a different effect on the EPL and ESCC risks. For the homozygous wild genotype CC subjects who have normal activity of MTHFR, high serum levels of vitamin B_2_ and B_12_ were significantly associated with reduced risk of EPL or ESCC. For the heterozygote CT, high serum levels of vitamin B_2_ and B_12_ showed the similar but weaker trends compared with the genotype CC. Vitamin B_2_ and B_12_ play the roles of cofactors for the enzymes MTHFR and methionine synthase. Insufficient available cofactors leads to folate becoming trapped as 5,10-methylenetetrahydrofolate or 5-methyltetrahydrofolate, whereas the generation of methionine is inhibited and the levels of homocysteine and related metabolites are increased because the remethylation of homocysteine to methionine intersects with this process of the folate cycle, but increased levels of homocysteine within the cell are reported to be toxic [11]. Additionally, the folate cycle is essential for purine and pyrimidine nucleotide synthesis and thus has a great role in the formation and stability of DNA, RNA and nucleoside triphosphates [37, 38]. Therefore, vitamin B deficiency in folate cycle can cause the altered expression of critical proto-oncogenes and tumor suppressor genes by negatively affecting DNA methylation, which may be an important step toward neoplasia [39].

It has consistently been demonstrated that vitamin B_2_ and B_12_ are essential for normal growth, development and physiological functions [11]. The combination of Table 2 and Fig. 1 show that compared with control group, EPL group had significantly poorer nutritional status of vitamin B_2_, and ESCC group had significantly poorer nutritional status of both vitamin B_2_ and B_12_. Previous study also indicated that a poor nutritional status and unbalanced diet were related to an elevated risk of cancer [40]. However, the possible roles of vitamin B_2_ and B_12_ in the etiology of ESCC are very complex, because they may have different impacts on this tumor development under different conditions at different stages of initiation, promotion and progression [41]. For example, preventing tumor initiation during the early stage of carcinogenesis, whereas promoting cancer development during the later stage in carcinogenesis (i.e., once precancerous lesions are established) can be possible [9, 42, 43]. Our study indicated that the interaction between the serum levels of vitamin B_2_ and B_12_ and the variant genotype TT was associated with both significant and non-significant tendencies for increased risk in the precancerous stage of esophageal carcinogenesis. The variant homozygotes TT for the C677T variant only has 30% MTHFR enzyme activity of the homozygous wild genotype CC, consequently the utilization rate of cofactors such as vitamin B_2_ and B_12_ is reduced in the folate cycle. Therefore, the MTHFR 677T allele may negate some of the protective effect of vitamin B_2_ and B_12_ in this way, and the corresponding impaired DNA methylation, synthesis and repair may be responsible for the development of precancerous lesions. In individuals with the homozygous wild genotype CC who had the lowest quartile of serum vitamin B_12_ level as reference, those with the homozygous mutation TT who had the highest quartile of serum vitamin B_12_ level had an tenfold increase in EPL risk (*p *< 0.05). It is possible to hypothesize that the subjects with TT genotype who had extremely low MTHFR enzyme activity may consume very low amount of vitamin B_12_ as cofactor in the folate cycle, thus the concentration of vitamin B_12_ in serum still remained high. Previous studies demonstrated that high intake of vitamin B_12_ can lead to increased level of the universal methyl donor S-adenosyl methionine and consequently result in the higher activity of DNA methyltransferase enzymes, which plays a role in the self-renewal of cancer stem cells and affect neoplasia and metastasis [44–46]. On the other hand, a population-based case–control study in Connecticut found the similar result and inferred that vitamin B_12_ can be a marker for consumption of animal-derived foods because vitamin B_12_ is derived exclusively from these foods such as red meat, which is associated with high risk of ESCC [47]. Additionally, B vitamins including folate, vitamin B_6_, B_9_ and B_12_ have been found to have a double-edged sword effect on cancer due to their dual role in carcinogenesis by possessing dual modulatory effects that are time and dose dependent [48–50]. As we know, the development of cancer typically has a very long latency period, thus the possibility that there might be preventive or harmful effects of B vitmians on cancer within the long period of time cannot be excluded [49].

However, the results so far in the field appear to differ significantly by individual. On the other hand, because of the limitation of the case–control study in establishing a causal association in this study, the low levels of vitamin B_2_ and B_12_ may also be a consequence, but not a risk factor for ESCC. It is possible that the ESCC cases are more likely to be on poor nutritional status due to cachexia, and tumor growth may result in metabolic disorder of vitamin B_2_ and B_12_ in ESCC cases. Thus further studies establishing the causal association and evaluating the mechanism are needed.

In this study, we have noted a high frequency of both p16 and p53 promoter methylation among both EPL and ESCC subjects. This finding is in line with previous studies on p16 and p53 methylation and ESCC [18, 19]. Here, the hypermethylation of p53 promoter was associated with silencing of protein expression of the gene. Because as an important tumor suppressor gene, p53 is essential for regulating cell division and preventing tumor formation, the silencing of it may be associated with the development of tumor and a higher risk of ESCC. In addition, p53 promoter methylation was also frequently found in variety of malignancies, such as breast carcinomas, gliomas, acute lymphoblastic leukemia, hepatocellular carcinomas and ovarian cancer [51–55]. Similarly, p16 silencing was also associated with the DNA methylation in the p16 promoter, which suggests that p16 silencing in ESCC may be a frequent event in the endemic region. A previous study has assessed the DNA methylation status in each sample based on the probe located in the p16 promoter CpG island, and reported that inactivation of p16 was present in 76% of both EAC and ESCC. However, the epigenetic silencing of p16 was frequent in EAC, whereas relatively rare in ESCC [56]. The inconsistency of the results between studies may differ in study regions, study design and study samples: firstly, Vietnamese patients were the only Asian population studied in the previous study, and other populations were from Eastern European and America continent. However, these populations are not able to represent rural population in China as they have completely different patterns of epidemiology, and the geographic distribution of ESCC varies greatly [2]; Secondly, the tested samples were different, such as blood samples and tissue samples. During the development of carcinogenesis, it has been found that epigenetic silencing of tumor suppressor genes by aberrant DNA methylation is an early major event [10, 18], but the interaction between aberrant DNA methylation and the MTHFR 677T allele cannot be ruled out. Our study demonstrated the gene–gene interaction between p16 and p53 methylations and MTHFR C677T polymorphism, suggesting that they may have an even greater role to play in cancer development than the single effect seen to date. After adjustment for potential confounding variables, the present study found that subjects carrying genotypes CT or TT and methylated genes p16 or p53 had high risks for EPL and ESCC, especially variant genotype TT. As reported previously, the activity of MTHFR is involved in the DNA methylation process, and MTHFR C677T polymorphism and folate status can interact in ways which affect DNA methylation status as well [17, 57]. Individuals carrying CT or TT genotype are more likely to have significant risk of DNA hypermethylation [10, 17]. However, so far it is still controversial about the role of MTHFR genetic polymorphisms because it may also have a dual role in the cancer development depending on different conditions including folate status, and the interaction between MTHFR C677T polymorphism and aberrant DNA methylation may vary from gene to another [17]. Despite decades of research on cancer-related genes, resulting in a staggering number of publications, researchers are only beginning to grasp the full complexity of the gene pathways and their intercations.

Based on our results, one of the important implications of this study was to detect the population who may be susceptible to ESCC by finding and applying eligible biomarkers, so as to improve the work of early diagnosis of ESCC. An exciting clue was obtained by the results that combining the detection of MTHFR C677T polymorphism and aberrant DNA methylation of p16 and p53 can find the individual who has extremely high risk of ESCC or EPL. However, this study also had limitation. It should also be pointed as the limitation of the case–control study in establishing a causal assocaition.

## Conclusions

In conclusion, this case–control study evaluated the association and interaction of epigenetics and genetics, serum vitamin B_2_ and B_12_ levels and the risks of EPL and ESCC in a high risk rural area in the Northern Jiangsu Province of China. Healthy controls were more likely to have higher levels of vitamin B_2_ and B_12_ than ESCC cases, and the MTHFR C677T genotype may modify the association between serum concentrations of vitamin B_2_ and B_12_ and the risks of EPL and ESCC. The gene–gene interaction suggested the possible crosstalk between the aberrant DNA methyaltion of either p16 or p53 and T alleles of MTHFR. In the future, further large-scale prospective studies on cancers are required to bring a more extensive understanding of DNA methylation, gene expression, dietary and environmental factors and their possible interactions in carcinogenesis, thus many novel and unexpected discoveries will be made, even leading to a reversal of earlier conclusions.

## Supplementary information

**Additional file 1: Figure S1.** Map of location of Huai’an District in China. Reprinted from Map of Huai’an by Maphill, April 2 2019, retrieved from http://www.maphill.com/china/jiangsu/huaian/maps/physical-map/Copyright 2013 by Maphill.

**Additional file 2: Figure S2. (a)** RFLP photograph of 3% agarose gel electrophoresis representing MTHFR C667T polymorphism. Lane 1 was characterized by single 233 bp representing wild genotype CC; lanes 2, 3, 4, 5 were characterized by 233, 176 and 57 bp representing heterozygote CT; lane 6 was characterized by 176 and 57 bp representing variant genotype TT; lane M represents DNA marker. **(b)** Photograph of 2% agarose gel electrophoresis representing results of methylation-specific PCR analysis for gene p16 and p53 by using both methylated (M) and unmethylated (U) specific primers. A, EPL case; B and C, ESCC cases; D, normal control; M, marker.

## Data Availability

All data generated or analysed during this study are included in this article. Due to our internal policy, raw data cannot be shared.

## Abbreviations

ANOVA: analysis of variance
CDC: center for disease control and prevention
CI: confidence intervals
EAC: esophageal adenocarcinoma
EC: esophageal cancer
EDETPEC: Early Diagnosis and Early Treatment Project of Esophageal Cancer
ELISA: enzyme-linked immunosorbent assay
EPL: esophageal precancerous lesion
ESCC: esophageal squamous cell carcinoma
MTHFR: methylenetetrahydrofolate reductase
MSP: methylation-specific PCR
OD: optical density
OR: odds ratios
PCR–RFLP: polymerase chain reaction-restriction fragment length polymorphism

## References

[1] Arnal MJD, Arenas ÁF, Arbeloa ÁL (2015). Esophageal cancer: risk factors, screening and endoscopictreatment in Western and Eastern countries. World J Gastroenterol.

[2] Abnet CC, Arnold M, Wei WQ (2018). Epidemiology of esophageal squamous cell carcinoma. Gastroenterology.

[3] Rustgi AK, El-Serag HB (2014). Esophageal carcinoma. N Engl J Med.

[4] Arnold M, Soerjomataram I, Ferlay J (2015). Global incidence of oesophageal cancer by histological subtype in 2012. Gut.

[5] Wang G, Abnet C, Shen Q (2005). Histological precursors of oesophageal squamous cell carcinoma: results from a 13 year prospective follow up study in a high risk population. Gut.

[6] Taylor PR, Abnet CC, Dawsey SM (2013). Squamous dysplasia—the precursor lesion for esophageal squamous cell carcinoma. Cancer Epidemiol Biomark Prev.

[7] Qiang Y, Li Q, Xin Y (2018). Intake of dietary one-carbon metabolism-related B vitamins and the risk of esophageal cancer: a dose-response meta-analysis. Nutrients.

[8] Xiao Q, Freedman ND, Ren J (2014). Intakes of folate, methionine, vitamin B6, and vitamin B12 with risk of esophageal and gastric cancer in a large cohort study. Br J Cancer.

[9] Ulrich CM, Potter JD (2007). Folate and cancer—timing is everything. JAMA.

[10] Chen J, Huang ZJ, Duan YQ (2011). Aberrant DNA methylation of P16, MGMT, and hMLH1 genes in combination with MTHFR C677T genetic polymorphism and folate intake in esophageal squamous cell carcinoma. Asian Pac J Cancer Prev.

[11] Rush EC, Katre P, Yajnik CS (2014). Vitamin B12: one carbon metabolism, fetal growth and programming for chronic disease. Eur J Clin Nutr.

[12] Matejcic M, de Batlle J, Ricci C (2017). Biomarkers of folate and vitamin B12 and breast cancer risk: report from the EPIC cohort. Int J Cancer.

[13] Hultdin J, Van Guelpen B, Bergh A (2005). Plasma folate, vitamin B12, and homocysteine and prostate cancer risk: a prospective study. Int J Cancer.

[14] Price AJ, Travis RC, Appleby PN (2016). Circulating folate and vitamin B12 and risk of prostate cancer: a collaborative analysis of individual participant data from six cohorts including 6875 cases and 8104 controls. Eur Urol.

[15] de Vogel S, Meyer K, Fredriksen A (2013). Serum folate and vitamin B12 concentrations in relation to prostate cancer risk—a Norwegian population-based nested case-control study of 3000 cases and 3000 controls within the JANUS cohort. Int J Epidemiol.

[16] Larsson SC, Giovannucci E, Wolk A (2006). Folate intake, MTHFR polymorphisms, and risk of esophageal, gastric, and pancreatic cancer: a meta-analysis. Gastroenterology.

[17] Wang J, Sasco AJ, Fu C (2008). Aberrant DNA methylation of P16, MGMT, and hMLH1 genes in combination with MTHFR C677T genetic polymorphism in esophageal squamous cell carcinoma. Cancer Epidemiol Biomarkers Prev.

[18] Das M, Sharma SK, Sekhon GS (2017). p16 gene silencing along with p53 single-nucleotide polymorphism and risk of esophageal cancer in Northeast India. Tumour Biol J Int Soc Oncodev Biol Med.

[19] Xu R, Wang F, Wu L (2013). A systematic review of hypermethylation of p16 gene in esophageal cancer. Cancer Biomark.

[20] Das M, Saikia BJ, Sharma SK (2015). p16 hypermethylation: a biomarker for increased esophageal cancer susceptibility in high incidence region of North East India. Tumour Biol.

[21] Shao Y, Tan W, Zhang S (2008). P53 gene codon 72 polymorphism and risk of esophageal squamous cell carcinoma: a case/control study in a Chinese population. Dis Esophagus.

[22] Zhao LJ, Zhao XL, Wu XM (2013). Association of p53 Arg72Pro polymorphism with esophageal cancer: a meta-analysis based on 14 case-control studies. Genet Test Mol Biomarkers.

[23] Piao JM, Kim HN, Song HR (2011). p53 codon 72 polymorphism and the risk of esophageal cancer: a Korean case-control study. Dis Esophagus.

[24] Hibi K, Taguchi M, Nakayama H (2001). Molecular detection of p16 promoter methylation in the serum of patients with esophageal squamous cell carcinoma. Clin Cancer Res.

[25] Lu Y, Zabihula B, Yibulayin W (2017). Methylation and expression of RECK, P53 and RUNX genes in patients with esophageal cancer. Oncol Lett.

[26] Huang GL, Wang SK, Su M (2013). Serum folate, MTHFR C677T polymorphism and esophageal squamous cell carcinoma risk. Biomed Environ Sci.

[27] Pan D, Su M, Zhang T (2019). a distinct epidemiologic pattern of precancerous lesions of esophageal squamous cell carcinoma in a high-risk area of Huai’an, Jiangsu Province, China. Cancer Prevent Res.

[28] Wang Z, Tang L, Sun G (2006). Etiological study of esophageal squamous cell carcinoma in an endemic region: a population-based case control study in Huaian, China. BMC Cancer.

[29] Ismail-Beigi F, Horton PF, Pope CE (1970). Histological consequences of gastroesophageal reflux in man. Gastroenterology.

[30] Weinstein WM, Bogoch ER, Bowes KL (1975). The normal human esophageal mucosa: a histological reappraisal. Gastroenterology.

[31] Dawsey SM, Lewin KJ, Liu FS (1994). Esophageal morphology from Linxian, China. Squamous histologic findings in 754 patients. Cancer.

[32] Park HJ, Yu E, Shim YH (2006). DNA methyltransferase expression and DNA hypermethylation in human hepatocellular carcinoma. Cancer Lett.

[33] Langevin SM, Lin D, Matsuo K (2009). Review and pooled analysis of studies on MTHFR C677T polymorphism and esophageal cancer. Toxicol Lett.

[34] Bailey LB, Gregory JF (1999). Polymorphisms of methylenetetrahydrofolate reductase and other enzymes: metabolic significance, risks and impact on folate requirement. J Nutr.

[35] Yang CX, Matsuo K, Ito H (2005). Gene-environment interactions between alcohol drinking and the MTHFR C677T polymorphism impact on esophageal cancer risk: results of a case-control study in Japan. Carcinogenesis.

[36] Ulvik A, Ueland PM, Fredriksen A (2007). Functional inference of the methylenetetrahydrofolate reductase 677 C > T and 1298A > C polymorphisms from a large-scale epidemiological study. Hum Genet.

[37] Sinclair KD, Allegrucci C, Singh R (2007). DNA methylation, insulin resistance, and blood pressure in offspring determined by maternal periconceptional B vitamin and methionine status. Proc Natl Acad Sci USA.

[38] Lillycrop KA (2011). Effect of maternal diet on the epigenome: implications for human metabolic disease. Proc Nutr Soc.

[39] Choi SW, Mason JB (2000). Folate and carcinogenesis: an integrated scheme. J Nutr.

[40] Kumar A, Kumari S, Poojary D (2014). Estimation of serum micronutrient levels and the possible risk of oral cancer and premalignancy. Int J Innov Res Sci Eng Technol.

[41] Siassi F, Ghadirian P (2005). Riboflavin deficiency and esophageal cancer: a case control-household study in the Caspian Littoral of Iran. Cancer Detect Prev.

[42] Ulrich CM (2007). Folate and cancer prevention: a closer look at a complex picture. Am J Clin Nutr.

[43] Smith AD, Kim YI, Refsum H (2008). Is folic acid good for everyone?. Am J Clin Nutr.

[44] Liu AY, Scherer D, Poole E (2013). Gene-diet-interactions in folate-mediated one-carbon metabolism modify colon cancer risk. Mol Nutr Food Res.

[45] Morita R, Hirohashi Y, Suzuki H (2013). DNA methyltransferase 1 is essential for initiation of the colon cancers. Exp Mol Pathol.

[46] Pathania R, Ramachandran S, Elangovan S (2015). DNMT1 is essential for mammary and cancer stem cell maintenance and tumorigenesis. Nat Commun.

[47] Mayne ST, Risch HA, Dubrow R (2001). Nutrient intake and risk of subtypes of esophageal and gastric cancer. Cancer Epidemiol Biomark Prev.

[48] Kim YI (2006). Folate: a magic bullet or a double edged sword for colorectal cancer prevention?. Gut.

[49] Zhang SM, Cook NR, Albert CM (2008). Effect of combined folic acid, vitamin B6, and vitamin B12 on cancer risk in women: a randomized trial. JAMA.

[50] Brasky TM, White E, Chen CL (2017). Long-term, supplemental, one-carbon metabolism-related vitamin B use in relation to lung cancer risk in the vitamins and lifestyle (VITAL) cohort. J Clin Oncol.

[51] Kang JH, Kim SJ, Noh DY (2001). Methylation in the p53 promoter is a supplementary route to breast carcinogenesis: correlation between CpG methylation in the p53 promoter and the mutation of the p53 gene in the progression from ductal carcinoma in situ to invasive ductal carcinoma. Lab Invest.

[52] Amatya VJ, Naumann U, Weller M (2005). TP53 promoter methylation in human gliomas. Acta Neuropathol.

[53] Agirre X, Vizmanos JL, Calasanz MJ (2003). Methylation of CpG dinucleotides and/or CCWGG motifs at the promoter of TP53 correlates with decreased gene expression in a subset of acute lymphoblastic leukemia patients. Oncogene.

[54] Pogribny IP, James SJ (2002). Reduction of p53 gene expression in human primary hepatocellular carcinoma is associated with promoter region methylation without coding region mutation. Cancer Lett.

[55] Chmelarova M, Krepinska E, Spacek J (2013). Methylation in the p53 promoter in epithelial ovarian cancer. Clin Transl Oncol.

[56] Kim J, Bowlby R, Mungall AJ (2017). Integrated genomic characterization of oesophageal carcinoma. Nature.

[57] Friso S, Choi SW, Girelli D (2002). A common mutation in the 5,10-methylenetetrahydrofolate reductase gene affects genomic DNA methylation through an interaction with folate status. Proc Natl Acad Sci USA.

