# Supplementary material for: MTHFR C677T genetic polymorphism in combination with serum vitamin B2, B12 and aberrant DNA methylation of P16 and P53 genes in esophageal squamous cell carcinoma and esophageal precancerous lesions: a case–control study
Source: Cancer Cell Int. 2019 Nov 12;19:288. doi: 10.1186/s12935-019-1012-x (PMC6852963; doi:10.1186/s12935-019-1012-x)
Supplement: Supplementary file 1 — Additional file 1: Figure S1. Map of location of Huai’an District in China. Reprinted from Map of Huai’an by Maphill, April 2 2019, retrieved from http://www.maphill.com/china/jiangsu/huaian/maps/physical-map/Copyright 2013 by Maphill. [file 12935_2019_1012_MOESM1_ESM.doc]

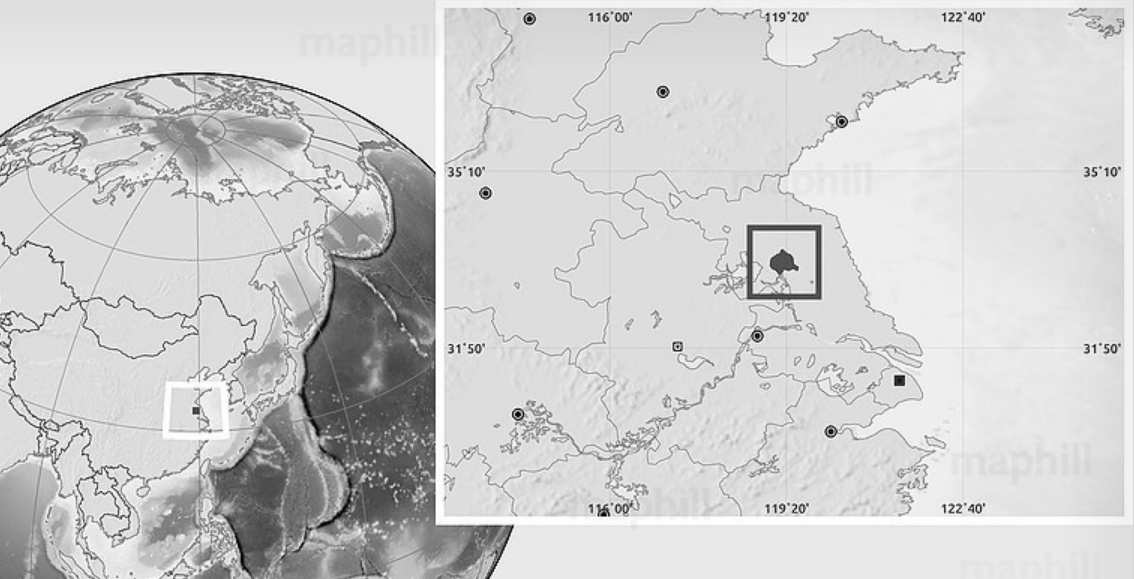


**Figure S1.** Map of location of Huai'an District in China. Reprinted from Map of Huai'an by Maphill, April 2 2019, retrieved from http://www.maphill.com/china/jiangsu/huaian/maps/physical-map/ Copyright 2013 by Maphill.
