# Supplementary material for: MTHFR C677T genetic polymorphism in combination with serum vitamin B2, B12 and aberrant DNA methylation of P16 and P53 genes in esophageal squamous cell carcinoma and esophageal precancerous lesions: a case–control study
Source: Cancer Cell Int. 2019 Nov 12;19:288. doi: 10.1186/s12935-019-1012-x (PMC6852963; doi:10.1186/s12935-019-1012-x)
Supplement: Supplementary file 2 — Additional file 2: Figure S2. (a) RFLP photograph of 3% agarose gel electrophoresis representing MTHFR C667T polymorphism. Lane 1 was characterized by single 233 bp representing wild genotype CC; lanes 2, 3, 4, 5 were characterized by 233, 176 and 57 bp representing heterozygote CT; lane 6 was characterized by 176 and 57 bp representing variant genotype TT; lane M represents DNA marker. (b) Photograph of 2% agarose gel electrophoresis representing results of methylation-specific PCR analysis for gene p16 and p53 by using both methylated (M) and unmethylated (U) specific primers. A, EPL case; B and C, ESCC cases; D, normal control; M, marker. [file 12935_2019_1012_MOESM2_ESM.doc]

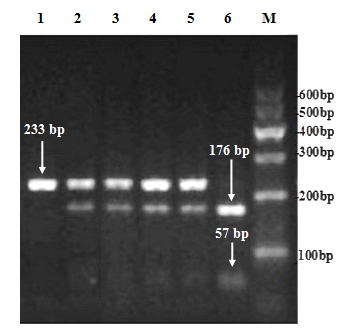


**(a)**


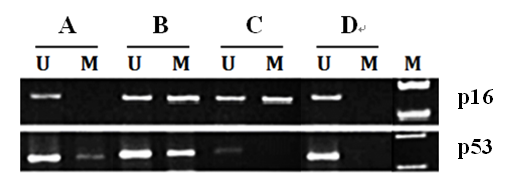


**(b)**

**Figure S2. (a)** RFLP photograph of 3% agarose gel electrophoresis representing MTHFR C667T polymorphism. Lane 1 was characterized by single 233 bp representing wild genotype CC; lanes 2, 3, 4, 5 were characterized by 233, 176 and 57 bp representing heterozygote CT; lane 6 was characterized by 176 and 57 bp representing variant genotype TT; lane M represents DNA marker.

**(b)** Photograph of 2% agarose gel electrophoresis representing results of methylation-specific PCR analysis for gene p16 and p53 by using both methylated (M) and unmethylated (U) specific primers. A, EPL case; B and C, ESCC cases; D, normal control; M, marker.
